# Energy at Origins: Favorable Thermodynamics of Biosynthetic Reactions in the Last Universal Common Ancestor (LUCA)

**DOI:** 10.3389/fmicb.2021.793664

**Published:** 2021-12-13

**Authors:** Jessica L. E. Wimmer, Joana C. Xavier, Andrey d. N. Vieira, Delfina P. H. Pereira, Jacqueline Leidner, Filipa L. Sousa, Karl Kleinermanns, Martina Preiner, William F. Martin

**Affiliations:** ^1^Department of Biology, Institute of Molecular Evolution, Heinrich Heine University Düsseldorf, Düsseldorf, Germany; ^2^Department of Functional and Evolutionary Ecology, University of Vienna, Vienna, Austria; ^3^Department of Chemistry, Institute of Physical Chemistry, Heinrich Heine University Düsseldorf, Düsseldorf, Germany

**Keywords:** origin of life, energetics, bioenergetics, metabolism, early evolution, biosynthesis, thermodynamics, last universal common ancestor

## Abstract

Though all theories for the origin of life require a source of energy to promote primordial chemical reactions, the nature of energy that drove the emergence of metabolism at origins is still debated. We reasoned that evidence for the nature of energy at origins should be preserved in the biochemical reactions of life itself, whereby changes in free energy, Δ*G*, which determine whether a reaction can go forward or not, should help specify the source. By calculating values of Δ*G* across the conserved and universal core of 402 individual reactions that synthesize amino acids, nucleotides and cofactors from H_2_, CO_2_, NH_3_, H_2_S and phosphate in modern cells, we find that 95–97% of these reactions are exergonic (Δ*G* ≤ 0 kJ⋅mol^−1^) at pH 7-10 and 80-100°C under nonequilibrium conditions with H_2_ replacing biochemical reductants. While 23% of the core’s reactions involve ATP hydrolysis, 77% are ATP-independent, thermodynamically driven by Δ*G* of reactions involving carbon bonds. We identified 174 reactions that are exergonic by –20 to –300 kJ⋅mol^−1^ at pH 9 and 80°C and that fall into ten reaction types: six pterin dependent alkyl or acyl transfers, ten S-adenosylmethionine dependent alkyl transfers, four acyl phosphate hydrolyses, 14 thioester hydrolyses, 30 decarboxylations, 35 ring closure reactions, 31 aromatic ring formations, and 44 carbon reductions by reduced nicotinamide, flavins, ferredoxin, or formate. The 402 reactions of the biosynthetic core trace to the last universal common ancestor (LUCA), and reveal that synthesis of LUCA’s chemical constituents required no external energy inputs such as electric discharge, UV-light or phosphide minerals. The biosynthetic reactions of LUCA uncover a natural thermodynamic tendency of metabolism to unfold from energy released by reactions of H_2_, CO_2_, NH_3_, H_2_S, and phosphate.

## Introduction

Between the first appearance of liquid water on the Earth roughly 4.3 billion years ago (Mojzsis et al., 2001) and the appearance of the first signs of life roughly 3.8 billion years ago (Rosing, 1999), simple spontaneous geochemical reactions gave rise to the enzymatically catalyzed reaction network of microbial metabolism: a highly organized set of specific organic reactions that provides the amino acids, nucleotides and cofactors to sustain ribosomal protein synthesis and growth. How metabolism arose is a keystone issue for understanding how the first microbes arose from the elements. It is a complex problem with many facets, several approaches to investigate the issue are current.

From the standpoint of theory, autocatalytic networks provide a useful framework for the study of metabolic origin (Kauffman, 1986; Hordijk and Steel, 2004). In autocatalytic sets, elements of the set can catalyze the synthesis of other elements of the set, potentially giving rise to molecular self-organization provided that a food source is supplied to drive the network forward (Hordijk et al., 2010). Autocatalytic sets are not purely theoretical objects because they can be identified in the metabolism of both modern cells and their inferred ancestors (Sousa et al., 2015; Xavier et al., 2020).

From the standpoint of individual reactions, inorganic catalysts have long been known to catalyze many metabolic reactions under laboratory conditions (Wächtershäuser, 1992; Huber and Wächtershäuser, 1997; Martin and Russell, 2007; Sousa et al., 2018). More recently, complex reaction sets approximating biochemical pathways (Muchowska et al., 2019, 2020) and in some cases even exactly retracing biochemical pathways (Preiner et al., 2020) have been reported, uncovering a natural tendency of numerous metabolic reactions to unfold in the presence of transition metal catalysis. From the computational standpoint, simulations have been widely employed to study metabolic origin, particularly network expansion algorithms. These have been shown to generate small molecule networks consisting of up to hundreds of compounds with properties that resemble metabolism, with the caveat that networks so generated are not manifest as natural pathways in modern cells (Goldford et al., 2017, 2019; Tian et al., 2019).

Independent of the methodological approach, current investigations of metabolic origin tend to start from the acetyl-CoA pathway of CO_2_ fixation (Fuchs and Stupperich, 1985; Fuchs, 2011) for a number of reasons. It is the only pathway of CO_2_ fixation (i) that is both linear and exergonic (Berg et al., 2010), (ii) that occurs in both bacteria and archaea (Berg et al., 2010; Fuchs, 2011), and (iii) that traces to the last universal common ancestor (LUCA) (Weiss et al., 2016). Its exergonic nature allows coupling of H_2_-dependent CO_2_ reduction to ion pumping and ATP synthesis, as in acetogens (Schuchmann and Müller, 2014) and methanogens (Thauer et al., 2008), strict anaerobes that obtain both their carbon and energy from the reduction of CO_2_ with H_2_. Organisms that use the acetyl-CoA pathway still inhabit H_2_-producing geochemical systems (Magnabosco et al., 2018; Smith et al., 2019), habitats that existed on the early Earth (Sleep et al., 2011). The first intermediate of the acetyl-CoA pathway, formate, is synthesized geochemically via abiotic reactions in modern hydrothermal systems (Lang et al., 2010; Schrenk et al., 2013), as are the endproducts of energy metabolism via the pathway in acetogens (acetate; Sherwood Lollar et al., 2021) and in methanogens (methane; Proskurowski et al., 2008). In carbon metabolism, the acetyl-CoA pathway generates pyruvate as the main product (Fuchs, 2011) via reactions that require 10 enzymes and cofactors, yet those enzymes can be replaced by simple hydrothermal minerals such as awaruite (Ni_3_Fe), which convert H_2_ and CO_2_ into formate, acetate and pyruvate overnight at 100°C in water (Preiner et al., 2020). Such findings connect the carbon and energy metabolism of acetogens and methanogens to spontaneous geochemical processes in H_2_-producing hydrothermal vents via the chemical reactions of the acetyl-CoA pathway (Martin, 2020).

Thermodynamic studies in geochemical systems also point to an origin of metabolism from H_2_ and CO_2_ in a hydrothermal setting, as the synthesis of amino acids (Amend and Shock, 1998) and even prokaryotic cell mass (Amend and McCollom, 2009) from H_2_, CO_2_ and NH_3_ is exergonic under the chemical conditions germane to H_2_-producing hydrothermal vents. However, calculating Δ*G* for a one-step geochemical reaction that converts H_2_, CO_2_ and NH_3_ into amino acids (Amend and Shock, 1998; Amend et al., 2013) does not begin to capture the thermodynamic landscape of metabolism, either modern or ancient, because the biosynthesis of amino acids and all other cell constituents involve the entry of H_2_, CO_2_, and NH_3_ at a very small number of very specific enzymatic reactions, followed by their distribution in activated form as hydride, organic carbon or amino moieties in highly connected networks of intermediate conversions. For example, over 20 distinct reactions are involved in the synthesis of either tryptophan or purines from H_2_, CO_2_, and NH_3_ (Kanehisa and Goto, 2000). Studies of thermodynamics at metabolic origin ideally need to address the thermodynamics of individual metabolic reactions as they are organized in modern cells or in the inferred ancestors thereof.

Our present investigation into metabolic origin is based on comparative physiology. Wimmer et al. (2021a) identified roughly 400 reactions that are used by bacteria and archaea to synthesize the amino acids, nucleotides and cofactors required for growth. Because these reactions are universal, they represent core biosynthetic metabolism in the last universal common ancestor (LUCA). As such, they can be seen as the endpoint of metabolic origin on the one hand and the starting point of physiological diversification on the other. Here we have updated this set of reactions, which we designate as the metabolic core, to include the two-enzyme reaction sequence of substrate level phosphorylation used by acetogens and some methanogens (Rother and Metcalf, 2004) as an acetyl-CoA dependent source of cytoplasmic (membrane independent) ATP synthesis. Although the acetyl-CoA pathway is not universal, having been replaced by many other autotrophic (Berg et al., 2010; Fuchs, 2011; Hügler and Sievert, 2011; Steffens et al., 2021) and heterotrophic (Schönheit et al., 2016) carbon assimilation pathways during evolution, it traces to LUCA (Weiss et al., 2016) and, like many of LUCA’s biochemical reactions (Sousa et al., 2018), is older than the enzymes that catalyze its reactions (Martin, 2020). Though the remaining chemical reactions of the core do not occur in all genomes, as auxotrophies arise recurrently in evolution, they are universal at the level of primary production, the process that has fueled all ecosystems from origins to today (Hamilton et al., 2016; Martin et al., 2018). However, the enzymes that catalyze the reactions of the core are not universal, such that the core cannot be identified through purely genomic comparisons because (i) reactions that arose post LUCA, in particular O_2_-dependent reactions (Dailey et al., 2017; Jabłońska and Tawfik, 2021), need to be filtered out (Wimmer et al., 2021a), (ii) because lateral gene transfers of recently arisen pathways have to be filtered out (Weiss et al., 2016), and (iii) because the enzymes that catalyze these reactions are often unrelated across the archaeal-bacterial divide (Sousa et al., 2013), suggesting independent origins of enzymatic pathways from LUCA en route to the last common ancestors of archaea (Williams et al., 2017) and bacteria (Xavier et al., 2021), respectively.

Despite many unknowns concerning the process of metabolic origin, one factor provides stringent constraint: The chemical reactions that comprised LUCA’s metabolism, and those from which it arose, were perforce exergonic, for without energy release, no reactions will take place. It has long been recognized that energy was required to promote reactions at metabolic origin, but the nature of that energy has been debated. Many possible environmental sources of energy at origins have been suggested, including pyrophosphate (PP_i_; Schramm et al., 1962), cyclic polyphosphates (Ozawa et al., 2004), reduced phosphorous minerals (Pasek, 2020), ultraviolet light (Patel et al., 2015), radioactive decay (Ebisuzaki and Maruyama, 2017), lightning (Ducluzeau et al., 2009), geochemical pyrite synthesis (Wächtershäuser, 1992), geochemical ion gradients (Russell and Cook, 1995), geoelectrical potential (Kitadai et al., 2021), bolide impacts (Ferus et al., 2015), and heat (Muller, 1995). Modern cells in nature, however, harness none of those environmental energy sources, they harness redox reactions instead (Mitchell, 1961; Thauer et al., 1977; Müller et al., 2018), and conserve energy for metabolic use in the chemically accessible currency of ATP (Decker et al., 1970) or reduced ferredoxin (Herrmann et al., 2008; Buckel and Thauer, 2013; Müller et al., 2018). The fact that only a fraction of core biosynthetic reactions entail ATP hydrolysis (Wimmer et al., 2021a) leads to a seldom formulated question: What drove the majority of LUCA’s metabolic reactions forward? We reasoned that ATP-independent biosynthetic reactions might themselves be a possible primordial energy source, one that would be particularly conducive to the formation of autocatalytic networks (Xavier et al., 2020). To investigate further, we polarized the core biosynthetic network of LUCA in the direction of cell synthesis and estimated the changes of Gibbs energy for each individual reaction using the component contribution method (Flamholz et al., 2012; Noor et al., 2013; Beber et al., 2021) to identify the nature of ATP-independent exergonic reactions endogenous to LUCA’s biosynthetic metabolism.

## Materials and Methods

### Biosynthetic Network

**FIGURE 1:**
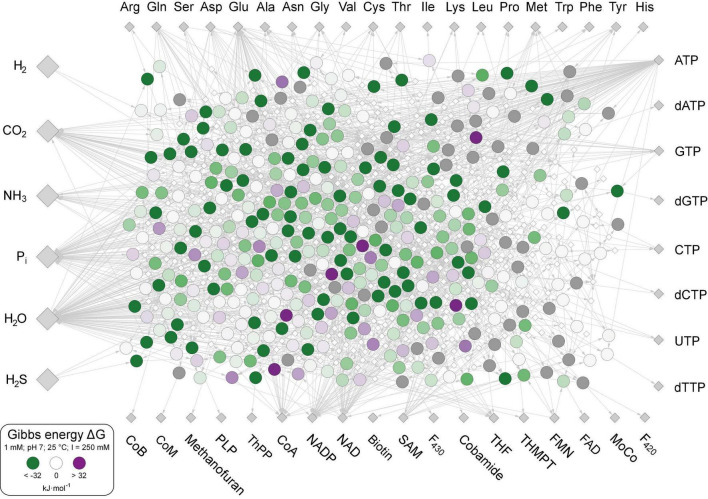
The biosynthetic core. Large gray diamonds: starting compounds (excluding trace elements). Small gray diamonds: products. White diamonds: intermediates. Large circles within the network: Reactions, values of Δ*G* calculated for 351 reactions at 25°C, pH 7 and 1 mM equal reactant and product concentration are indicated by large circles and colored according to color scale at lower left. The 51 reactions for which no value of Δ*G* could be calculated are indicated with dark gray shading. Reactions and values of Δ*G* given in Supplementary Table 3.

The 402 metabolic reactions comprising the core were manually polarized in the direction of cell synthesis (Wimmer et al., 2021a; Supplementary Table 1). Reactions of the acetyl-CoA pathway in the CO_2_ fixing reductive direction (Fuchs, 2011) [the archaeal pathway is missing in The Kyoto Encyclopedia of Genes and Genomes (KEGG)], gluconeogenesis (Say and Fuchs, 2010), the reverse citric acid cycle (Steffens et al., 2021) and the pentose phosphate pathway generate most key intermediates. No anaerobic synthesis was available in KEGG (the standard database for microbial metabolic pathways; Kanehisa and Goto, 2000) for dimethylbenzimidazole, 2-phospholactate and flavins. Three cofactors (CoB, CoM, and F_430_) that are not required in biosynthesis but are essential for ATP synthesis in methanogenic archaea (Thauer et al., 2008) are included in the core. The rare amino acids selenocysteine and pyrrolysine were not included, nor were modified amino acids in proteins as cofactors, including pyruvoyl enzymes. Reactions were obtained from KEGG (Kanehisa and Goto, 2000), version December 2020, excluding degradation reactions and oxygen-dependent reactions (Wimmer et al., 2021a), including H_2_-dependent substrate level phosphorylation (Martin and Thauer, 2017), ferredoxin:NAD(P)H interconversion, and H_2_-dependent CO_2_ reductase (Schuchmann and Müller, 2014). Of the 18 cofactors in Figure 1, 10 are required by the acetyl-CoA pathway in archaea and bacteria from H_2_ and CO_2_ to pyruvate (Fuchs and Stupperich, 1985; Martin, 2020). The biosynthetic pathway to iron-guanylylpridinol, required for H_2_-dependent methenyl H_4_MPT reduction in methanogenesis under nickel limitation (Huang et al., 2020), is not represented in KEGG and missing in the network, leaving only two entry points of H_2_ into metabolism via ferredoxin-reducing hydrogenases (Huang et al., 2020) and H_2_-dependent CO_2_ reductase (Schuchmann and Müller, 2014). Except biotin, the compounds clockwise from Trp to methanofuran in Figure 1 contain at least one aromatic ring. Aromatic ring forming reactions in the core entail five rings in amino acids, six in nucleoside bases, seven in pterins (two shared and five specific), eight in tetrapyrroles (four in pyrrole formation and four leading to F_430_ and cobalamin), two for methanofuran, one each for thiamine, pyridoxal, and pyridine dinucleotides. Each aromatic compound requires a ring formation reaction plus two non-aromatic rings in biotin and one each in ribose and proline. Modern chemolithoautotrophs live from the components shown on the left in Figure 1 plus trace elements (Magnabosco et al., 2018; Smith et al., 2019), growing on biotic H_2_ from fermentations (Wolfenden, 2011) or abiotic H_2_ from hydrothermal systems (Schrenk et al., 2013; Dick, 2019; Lang and Brazelton, 2020).

### Estimation of Gibbs Energy for Individual Reactions

A few words are needed concerning the component contribution (or group contribution) method. Traditionally, biochemists determine the change of Gibbs energy, Δ*G*, in a physiological reaction by measuring the concentrations of reactants and products in the presence of the enzyme. The change in Gibbs energy Δ*G* for the reaction A + B ⇌ C + D is obtained from the equation:


(1) ΔG=ΔG°+′RTln[C]⁢[D][A]⁢[B]

Where R is the gas constant, T is the temperature in Kelvin and [A], [B], [C], and [D] are the molar concentrations (more precisely activities) of reactants and products forming the reaction quotient. Δ*G’* is the change of free standard enthalpies during reaction in water at physiological pH 7, 25°C, 1 M molar concentrations and 1 atm gas pressure. If H^+^ is involved in the reaction, its activity is 1 in eq. (1) at pH 7. If water is involved in the reaction, its activity in eq. (1) is also 1 because Δ*G*’ is obtained from measurements in water and the water concentration in water as the solvent does not change appreciably by reaction water.

At equilibrium, Δ*G* = 0 (no net driving force and therefore no change of reactant and product concentrations anymore), resulting in:


(2) 0=ΔG°+′RTln[C]e⁢q⁢[D]e⁢q[A]e⁢q⁢[B]e⁢q

(3) ΔG°=′-RTln[C]e⁢q⁢[D]e⁢q[A]e⁢q⁢[B]e⁢q=-RTlnK′

Therefore, Δ*G*°’ can be obtained from the reactant and product concentrations measured at reaction equilibrium in water at pH 7. *K’* is the equilibrium constant at pH 7. The increments used in the component contribution method to obtain Δ*G*°’ derive their values from measurements of *K’* in water, hence the activity of water is already taken into account in Δ*G*°’ and can be set to 1 in the reaction quotient. At physiological conditions, concentrations are generally different from 1 M and eq. (1) with the reaction quotient term is used to calculate the Gibbs energy Δ*G’*. For clarity, we manually polarized the reactions toward synthesis by writing the KEGG reactions from left to right such that the flux of carbon and nitrogen starts from CO_2_ and NH_3_ and proceeds within the KEGG pathways in the direction of amino acid, nucleotide and cofactor synthesis. To estimate Δ*G* under nonequilibrium conditions, unequal reactant to product concentration ratios were inserted into in the reaction quotient for the polarized reaction, see below.

For many reactions catalogued in large biochemical databases such as KEGG (Kanehisa and Goto, 2000) the equilibrium concentrations are not known or not readily obtained, but the value of Δ*G’* can still be estimated using the component contribution method, which is based on the group contribution method originally developed by Benson (1968) to study the equilibria of chemical reactions in the gas phase and later adapted by Alberty (1998) and others to the study of aqueous reactions. It is an indirect method for estimating the position of the equilibrium in a reaction based on the thermodynamic contributions of the moieties in the compounds in question (Jankowski et al., 2008). In this paper we will use Δ*G’* to indicate 1 M reactant and product concentrations and 1 bar pressure for gasses at 25°C, in the strict sense. When we refer to conditions that deviate from Δ*G’*, for example different temperatures or different reactant and/or product concentrations, we use the generic term Δ*G*, whereby its parameters are then unambiguous by context.

Gibbs energies were calculated using eQuilibrator API (Flamholz et al., 2012; Beber et al., 2021) version 0.4.1 under Python v. 3.6.7 which bases its estimates on the component contribution method (Noor et al., 2013). eQuilibrator is widely used in biochemical and genome-based investigations, inter alia because it is capable of operating with reactions and compounds in the KEGG database. To cross check the current set, we compared values obtained using eQuilibrator to those determined by the traditional biochemical method for core carbon metabolism (Supplementary Table 2; Fuchs, 2011). As in earlier studies (Alberty, 1998; Jankowski et al., 2008; Flamholz et al., 2012), the agreement was good, usually within a few kJ⋅mol^−1^, indicating that the method delivers useful estimates.

**FIGURE 2:**
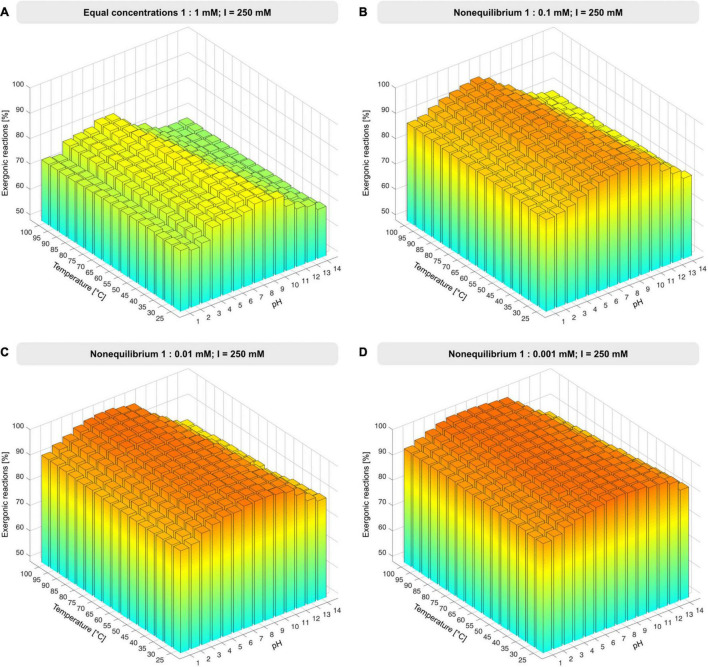
Proportion of exergonic reactions under nonequilibrium conditions. **(A)** At constant ionic strength, I, and reactant concentration of 1 mM, the concentration of products was decreased from 1 mM to 1 μM **(B–D)**, Δ*G* was calculated for the 351 reactions that deliver a value of Δ*G* across the pH and temperature range and the proportion of exergonic reactions was plotted for each condition set (see also Supplementary Figure 1 and Supplementary Table 3).

Unless otherwise specified, environmental conditions were simulated by varying the pH from 1 to 14 in increments of 1 and temperature from 25 to 100°C in increments of 5°C at constant ionic strength of 250 mM, Mg^2+^ concentration fixed to 3 mM, and reactant concentrations set to 1 mM. Nonequilibrium conditions were simulated by altering the reactant to product ratio from 1:1 to 1:0.1 mM, 1:0.01 mM, 1:0.001 mM, 1:0.0001 mM and 1:10 mM (Figure 2, Supplementary Figure 1, and Supplementary Table 3). Atomic balancing was checked prior to calculation, such that Δ*G* was only calculated for balanced reactions, excluding partial reactions. For 351 reactions Gibbs energy was calculable, for the remaining 51 reactions Δ*G* calculation failed due to involvement of KEGG compounds undefined in the eQuilibrator database, compounds having ambiguous structures, or unbalanced reactions.

Even though the reactions of biosynthetic metabolism are interconnected, we can consider each reaction individually with regard to its change in free energy in the biosynthetic direction, because the value for change of free energy for a given enzymatic reaction results from the physicochemical properties of its reactants and products under the specified conditions as in eq. (2). A directed metabolic network representing the 402 reactions was created in simple interaction format (sif). The bipartite graph was drawn with CytoScape (Shannon et al., 2003) v. 3.8.0. Reaction nodes and compound nodes were labeled as indicated in Figure 1.

### Substitution of Biochemical Reductants With Hydrogen

**FIGURE 3:**
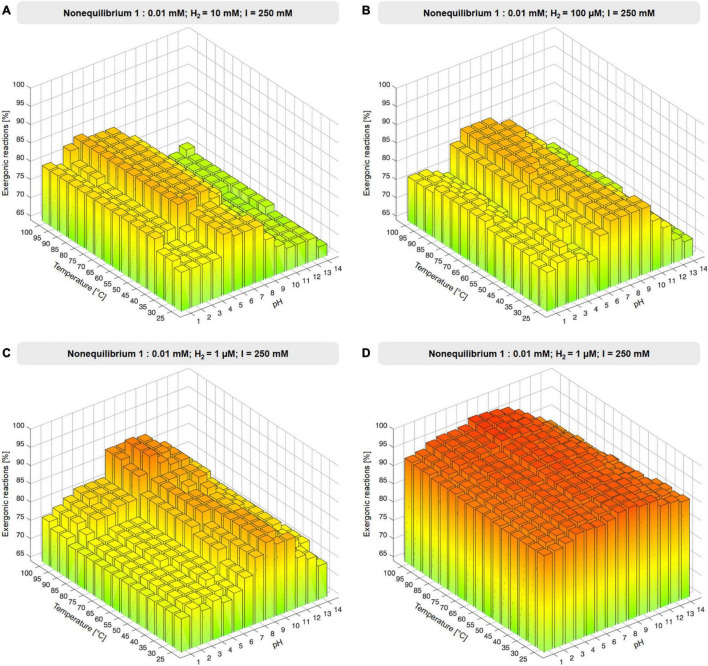
Effect of environmental H_2_. **(A–C)** Proportion of exergonic reactions at 1 μM, 100 μM, 10 mM H_2_ and 1:0.01 nonequilibrium at constant ionic strength I expressed as % of the 67 redox reactions of the core that yield values of Δ*G*. **(D)** Proportion of exergonic reactions at 1 μM H_2_ and 1:0.01 nonequilibrium expressed as % of 353 reactions for which values of Δ*G* were obtained (see also Supplementary Figure 3 and Supplementary Table 4).

To investigate the influence of environmental H_2_ in the 73 reactions involving biochemical reductants, NAD(P)H, reduced ferredoxin and reduced flavodoxin were replaced with H_2_, generating a reduced product and protons in the balanced equation (reaction equations are given in Supplementary Table 4), simulating H_2_ as a reductant present in an environmental setting. Ferredoxin:NADH oxidoreductase and ferredoxin reducing hydrogenase reactions were excluded from H_2_ substitution because H_2_ would have appeared on both sides of the reaction. Gibbs energies were calculated as for the altered set. In the substituted set, two additional reactions (353 total) yielded a value for Δ*G*, 49 did not. The compound concentration ratio was set to nonequilibrium 1:0.01 mM with fixed H_2_ reactant and product concentrations 1 μM, 10 μM, 100 μM, 1 mM, 10 mM, and 100 mM (Figure 3, Supplementary Figure 2, and Supplementary Table 4). The influence of ionic strength, *I*, was probed by altering *I* from standard 250 mM to 2.5 mM, 25 mM, 2.5 M, and 0 M under a nonequilibrium concentration ratio of 1:0.01 mM and with H_2_ fixed to 1 μM (see Supplementary Figure 3 and Supplementary Table 5). For all calculations, reactions are classified as exergonic if Δ*G* ≤ 0.

### Metal Catalyzed NAD^+^ Reduction With H_2_

NAD^+^ solution (3 mM) was prepared in a phosphate buffer at pH 8.5. Both glass reaction vials containing 4 ml NAD^+^ solution (no catalyst) and vials containing 4 ml NAD^+^ solution and nickel (Alfa Aesar) and iron powder (Alfa Aesar) as solid phase catalysts, added as 26 mg Fe plus 28 mg Ni powder per ml solution, were placed in a stainless-steel reactor (Berghof). The vials were closed with PTFE septum lids which were penetrated with syringe needles (Sterican) to ensure the reaction gas could enter the vials. The closed reactor was pressurized with 5 bar of hydrogen gas and heated up to 40°C for a total of 4 h. After depressurizing the reactor, samples were transferred to 2 ml Eppendorf tubes, centrifuged for 15 min at 13,000 rpm (Biofuge fresco, Heraeus) and the supernatant was collected to spectrophotometrically observe NADH synthesis (characteristic maximum absorbance at 339 nm; Cary 3500 UV-Vis, Agilent) (see Supplementary Figure 4). For convenience, conversion tables relating H_2_ partial pressures and H_2_ concentrations in water at different temperatures are given in Supplementary Table 6.

### Energetics of Amino Acid Synthesis

**FIGURE 4:**
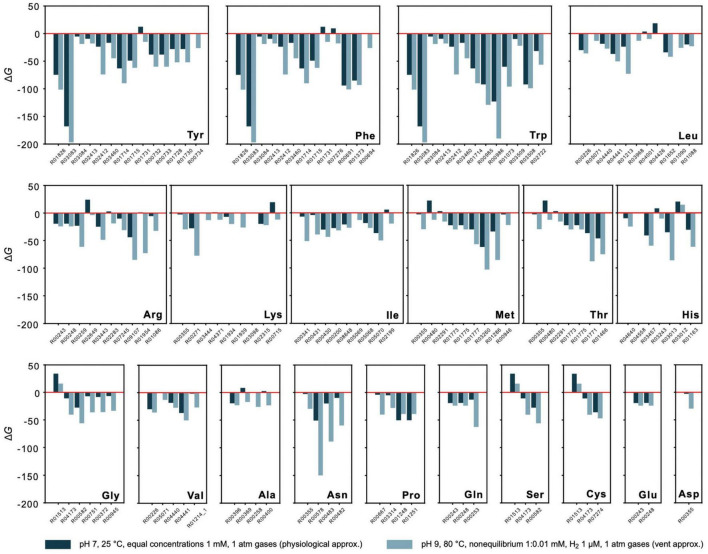
Energetics of amino acid synthesis. Gibbs energies Δ*G* were calculated for reactions involved in the synthesis of the 20 canonical amino acids. Only reactions yielding a value for Δ*G* are plotted. Values of Δ*G* are indicated for physiological (dark blue) and vent conditions (light blue) as defined in the figure. Underlying synthesis pathways are listed in Supplementary Table 7. Only pathways starting from key intermediates pyruvate, oxalacetate, 2-oxoglutarate, phosphoenolpyruvate, 3-phosphoglycerate, and C5 sugars (as proposed in Martin, 2020) are examined. Additional synthesis alternatives from KEGG are not taken into consideration.

Energetics of synthesis pathways for the 20 canonical amino acids consisting of KEGG reactions starting from key intermediates pyruvate, oxalacetate, 2-oxoglutarate, phosphoenolpyruvate, 3-phosphoglycerate, and C5 sugars (Martin, 2020; Supplementary Table 7 and Figure 4) were analyzed. The pathways, when expressed as linear sets of reactions, are detached from the biosynthetic core network by the removal of edges. Alternative pathway branches and reactions are indicated by numbers, for example 2.1 corresponds to the first reaction in the second pathway alternative. Gibbs energies for 1 mM reactant and product concentrations, pH 7 and 25°C are given in Supplementary Table 3 and for vent-like conditions (nonequilibrium 1:0.01 mM, pH 9 and 80°C) in Supplementary Table 4.

## Results

### Thermodynamics in the Metabolism of the Last Universal Common Ancestor

Theories of autotrophic origin posit that the first free living cells grew from CO_2_ and inorganic compounds without the help of light (Mereschkowsky, 1910; Fuchs and Stupperich, 1985; Wächtershäuser, 1992; Fuchs, 2011). For such chemolithoautotrophic cells to arise at a specific environmental site, the reactions underpinning their origin, that is, the overall set of reactions that synthesize the cell needs to be exergonic and no individual reaction should be so endergonic as to block the reaction network under physiological conditions. The source of energy that allows those reactions to go forward is of interest here. The synthesis of the amino acids, nucleotides and cofactors germane to life from H_2_, CO_2_, NH_3_, H_2_S, and P_i_ requires only 402 reactions (Wimmer et al., 2021a; Supplementary Table 1) which are listed in KEGG (Kanehisa and Goto, 2000). We polarized those reactions so that carbon flux through each reaction proceeds from H_2_ and CO_2_ in the direction of monomer synthesis. We then employed the component contribution method (Noor et al., 2013) to estimate the change in Gibbs energy, Δ*G*, for the 402 reactions in the biosynthetic direction (see section “Materials and Methods”).

The set of 402 polarized reactions in KEGG format contained 51 entries that yielded no value of Δ*G* because one or more reactants are poorly defined or have ambiguous structures, that is, they were not among the underlying data with which eQuilibrator works (see section “Materials and Methods”). The remaining 351 reactions yield thermodynamic estimates, providing a very broad sample for changes in Δ*G*, covering 87% of reactions in the core (see Supplementary Table 3). We started with the simple case of all reactants (compounds on the left side of reactions) and products (right side) at 1 mM concentration, a value well within the 1 μM to 10 mM range of metabolite concentrations in *Escherichia coli* during exponential growth (Bennett et al., 2009) to examine the effect of pH and temperature regarding metabolic origins under hot (Stetter, 2006) vs. cold (Miyakawa et al., 2002) or acidic (Wächtershäuser, 1988) vs. alkaline (Martin and Russell, 2007) conditions. Roughly 77% of core reactions are exergonic at pH 6-7, with temperature exerting little effect (Figure 2A). Note that the component contribution method does not obtain values for Δ*G* as a function of temperature, and that temperature effects are considered by the reaction quotient (see eq. (1) in section “Materials and Methods”).

### Nonequilibrium Conditions

Metabolism in cells is a connected series of far from equilibrium reactions in which reactants continuously react to products at every step (Decker et al., 1970; Battley, 1987; Dai and Locasale, 2018), whereby the products of one reaction become the reactants of the next in succession. As it concerns calculations of thermodynamic values, this presents a stark difference to geochemical thermodynamics, where one step reactions are the rule, for example balanced single step reactions for the synthesis of amino acids from H_2_, CO_2_, and NH_3_ (Amend and Shock, 1998, 2001). In the context of metabolic origin, the process to model concerns a situation in which compounds supplied by the environment (H_2_, CO_2_, and NH_3_ for example) react to generate products that do not initially exist (Martin and Russell, 2007), such as formate and pyruvate (Preiner et al., 2020) or amino acids. In a hydrothermal vent context, such compounds can either react further, or be eluted from their site of synthesis via hydrothermal effluent by convection and/or thermal or concentration diffusion. In cells, the products can either react further, or be excreted as an end product, generating steady state equilibrium (German: *Fließgleichgewicht*), or they can be converted to biological polymers—proteins, sugars, nucleic acids—exiting the metabolic network as cell mass. In acetogens, for example, roughly 24 molecules of CO_2_ are converted to acetate as an end product for every atom of carbon that is incorporated into cell mass (Daniel et al., 1990). We designate the situation of higher reactant concentrations relative to product concentrations as nonequilibrium conditions.

When examined using the component contribution method, the effect of nonequilibrium conditions is large. Increasing the product concentration 10-fold relative to reactant concentrations renders most reactions of the core endergonic (Supplementary Figure 1B). This is because many reactions in metabolism are close to equilibrium in terms of Δ*G*, with every 10-fold reduction in product concentration relative to reactant concentration corresponding to a change in Δ*G* of –5.7 kJ⋅mol^−1^ at 25°C (Walsh et al., 2018) for reactions having equal stoichiometric coefficients of reactants and products. Increasing product concentrations shows that the reactions of the core have little tendency to run backward (Supplementary Figure 1B), which is in line with the concept of autotrophic origins (Fuchs, 2011).

Lowering the concentration of products relative to reactants approximates the situation in an environmental setting in which H_2_, CO_2_, H_2_S, NH_3_, and phosphate (Figure 1) are continuously supplied in roughly constant amounts, while the products of reactions are allowed to react further or removed by flow processes. To model nonequilibrium conditions, we reduced the product concentrations in steps of 10-fold change relative to reactants (Figures 2B-D and Supplementary Figure 1). At 100-fold less product than reactant, 98% of core reactions become exergonic (Figure 2C), with marginal increase at higher ratios and no marked effect of temperature except at very high pH.

Regardless of the specific environment within which LUCA arose, the reactions fueling the synthesis of its building blocks underwent a transition during the origin of metabolism: Reactions that were initially either uncatalyzed or catalyzed by substances in the environment eventually came to be catalyzed by cofactors and enzymes encoded by genes. During that transition, it is possible, and cannot be excluded, that some or many of the chemical reactions themselves might have changed. But it is also possible, and cannot be excluded, that the reaction set remained essentially the same, as in the example of the acetyl-CoA pathway (Preiner et al., 2020) and reverse citric acid cycle (Muchowska et al., 2020). In that case, only the nature of the catalysts changed from inorganic to organic, adding specificity and rate to preexisting reactions that tend to occur anyway.

Because the core constitutes a minimal set of enzymatic reactions required for the synthesis of amino acids, nucleotides and cofactors, it contains neither a rotor stator ATPase, nor cytochromes, quinones, or even membrane-associated reactions. Although the rotor stator ATPase is as universal in cells as the ribosome itself, and was present in LUCA (Weiss et al., 2016), is not essential for the biosynthetic core to operate. Net ATP synthesis can be derived within the core from substrate level phosphorylation via acetate synthesis from H_2_ and CO_2_ in soluble reactions, similar to the situation of *Methanosarcina mazei* growing on CO (Rother and Metcalf, 2004). Also note that we are considering each reaction individually, not as a system of interconnected reactions set in series, in which case reactant concentrations would approach zero under nonequilibrium conditions. We are not querying the extent to which the overall balanced one-step reactions from H_2_, CO_2_, and NH_3_ to the individual amino acids, bases and cofactors are energy releasing, which for amino acids and nucleotides is known to be the case under the conditions of H_2_-producing hydrothermal vents interfacing with ocean water (Amend and McCollom, 2009). Instead, we are investigating the exergonic nature of the individual reactions in LUCA’s biosynthetic pathways, as they are manifest in modern enzymatic reactions, which are intensely interconnected in a metabolic network (Figure 1), applying the same concentration gradient to each, so that the individual chemical reactions underlying energy release within the network, as opposed to energy release for the network as a whole as in the energetics of growth (Battley, 1987; Hansen et al., 2009), can be identified.

**FIGURE 5:**
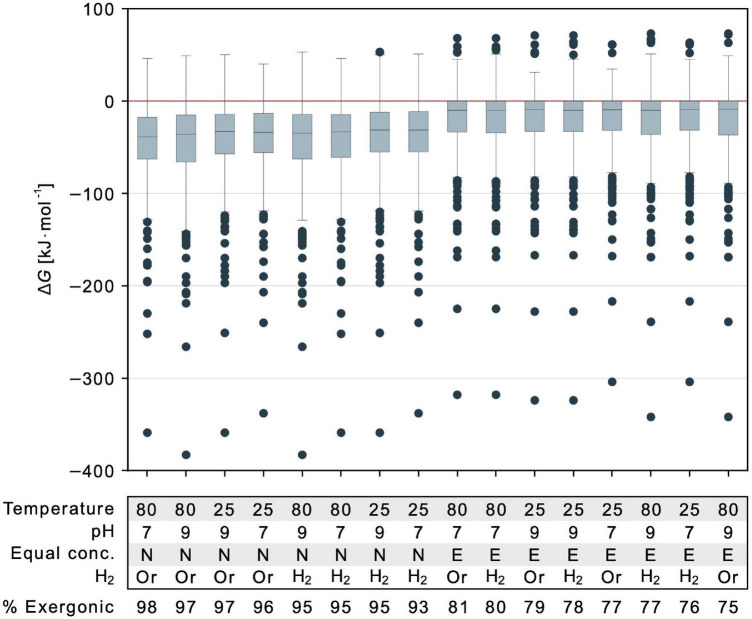
Energetic effect of temperature, pH, reductants and reactant to product concentration ratios. Gibbs energy is indicated for 351 calculable reactions that deliver a value of Δ*G* for different parameter combinations. Values for each parameter of each calculation are specified in the table below the figure and varied with respect to physiological and vent conditions. Temperature is described in degree Celsius. Equal conc.: *E* indicates all concentrations set to 1 mM, *N* indicates nonequilibrium 1:0.01 mM reactant to product ratio. Retention of organic reductants (NADH, NADPH, flavodoxin_red_, ferredoxin_red_) is indicated as *Or* while the replacement of these organic reductants with hydrogen is marked by *H*_2_. Note that in this case, two additional reactions yield a value for Δ*G* (353 reactions). Proportions of exergonic reactions (Δ*G* ≤ 0 kJ⋅mol^–1^) across the biosynthetic core are listed below the table. In each boxplot, the horizontal line indicates the median Δ*G* among calculable reactions. The colored boxes represent the interquartile range (IQR) with Δ*G* within quartile 1 (Q1, median of lower half of the data) and quartile 3 (Q3, median of upper half of the data). The range bars mark the minimum (Q1 – 1.5⋅IQR) and maximum (Q3 + 1.5⋅IQR) value of the data excluding any outliers. Outliers are indicated by individual dots and do not fall into the defined range between minimum and maximum.

The finding that 98% of the reactions in the core that deliver a value of Δ*G* using the component contribution method are exergonic under nonequilibrium conditions starting from H_2_ and CO_2_, with 100-fold less product than substrate, is noteworthy. It also reminds us that the reactions of metabolism as they operate in modern cells are generally exergonic, otherwise metabolism would not run. Yet even with equal substrate and product concentrations, on average 78% of the reactions in the core are exergonic under the conditions sampled here (Figure 5). As an caveat, many enzymatic reactions in the core might not go forward under prebiotic conditions for lack of suitable catalysts, for reasons of inhibitory inorganic compounds, due to substrate sequestration on surfaces, or for other reasons. Favorable thermodynamics are thus a necessary but not sufficient condition for the emergence of metabolism. We also note that our study addresses only monomer synthesis, not polymerization reactions. Notwithstanding, the present findings indicate that there is a natural thermodynamic tendency for the reactions of LUCA’s biosynthetic network to unfold from H_2_, CO_2_, NH_3_, H_2_O, and P_i_. This is not self-evident, because it introduces the possibility that the energy needed at the origin of metabolism simply stemmed from within metabolism itself, as opposed to some external source.

### The Effect of Environmental H_2_

At the very onset of the process that gave rise to LUCA’s metabolism, it is reasonable to assume there were no preformed organic redox cofactors in supply in the environment, as these are products of organic synthesis. Microbiologists have, however, long held that reduced low potential FeS centers such as those in ferredoxin were the source of reducing power in the early stages of biochemical evolution (Eck and Dayhoff, 1966; Hall et al., 1971). In line with that view, all hydrogenases in modern chemolithoautotrophs that use H_2_ as a reductant reduce FeS clusters, with only one known exception, the Fe hydrogenase of methanogens that transfers electrons from the active site of the iron-guanylylpyridinol (FeGP) cofactor directly to F_420_, generating F_420_H_2_ without the involvement of FeS or other intermediate electron carriers (Huang et al., 2020).

Hydrogen gas is also the source of electrons for chemolithoautotrophic archaea and bacteria that synthesize ATP by reducing CO_2_ (Thauer et al., 1977; Fuchs, 2011; Schuchmann and Müller, 2014; Preiner et al., 2020). In modern geological environments that generate abiotic hydrogen (Schrenk et al., 2013), H_2_ is synthesized in amounts that generate midpoint potentials on the order of –700 to –900 mV (Boyd et al., 2020), more than sufficient to substitute for known biochemical reductants such as NAD(P)H or reduced ferredoxins (Supplementary Table 6). The very low midpoint potentials come from an interplay of two factors: serpentinization generates H_2_ in a geochemical process that also generates metal hydroxides such as Mg(OH)_2_, which in turn generate alkalinity. Alkaline solutions foster the release of protons from H_2_ via heterolytic cleavage, leading to the release of electrons onto suitable acceptors. Some modern microbes that inhabit such H_2_-rich alkaline environments even appear to lack known hydrogenase enzymes (Suzuki et al., 2018), suggesting that there might be alternative or bypass entry points for H_2_ into their metabolism. To investigate the effect of environmental redox potential on the thermodynamics of the biosynthetic core, we replaced biological reductants by the environmental source of electrons in CO_2_-reducing autotrophs, H_2_, in all reactions of the core. This captures the thermodynamic effect of an environmental redox buffer, but entails the premise that mineral catalysts naturally occurring in hydrothermal vents (Fontecilla-Camps, 2019) can readily replace hydrogenases and ferredoxin to reduce the main biochemical hydride carrier, NAD(P)^+^, with H_2_. To that end, we tested H_2_-dependent NAD(P)^+^ reduction in the laboratory using simple transition mineral catalysts. The reaction is facile under hydrothermal conditions (Supplementary Figure 4).

As an environmental parameter, H_2_ reactant and product concentrations must be equal. This impacts redox reactions of the core under nonequilibrium conditions. The core encompasses 73 redox reactions involving NAD(P)H, flavins, or ferredoxin. Reduced cofactors occur on the left in 48 reactions and on the right in 27 (Table 1). We replaced biochemical reductants on both sides of the reactions with H_2_ at concentrations corresponding to an *E*_0_ of –600 to –800 mV at pH 10 around 100°C (Supplementary Table 6). For the reactant:product ratio of 1:0.01 mM under nonequilibrium conditions, we adjusted the H_2_ concentration on both sides of the reaction from 1 μM to 100 mM (Figure 3, Supplementary Figure 2, and Supplementary Table 4). For reactions in which H_2_ is a reactant, large H_2_ concentrations are favorable, whereas for reactions where H_2_ is product, low H_2_ concentrations are favorable. *Vice versa*, for reactions where H_2_ is reactant and H^+^ is product, high pH is favorable.

**TABLE 1:** Most frequent reactants and products in the core.

	Reactant						Product			
Compound	Left	Right	Physiological^a^		Vent^b^		Physiological^a^		Vent^b^	
			Δ*G* ≤ 0	Δ*G* > 0	Δ*G* ≤ 0	Δ*G* > 0	Δ*G* ≤ 0	Δ*G* > 0	Δ*G* ≤ 0	Δ*G* > 0
H_2_O	71	49	51	15	64	2	35	10	43	2
H^+^	48	31	33	7	37	3	18	10	26	2
ATP	77	2	55	14	69	0	2	0	2	0
P_i_	2	64	0	2	2	0	54	5	59	0
ADP	3	54	3	0	3	0	36	12	48	0
CO_2_	12	37	3	8	10	1	34	0	34	0
Glutamate	26	18	14	10	23	1	16	1	17	0
NAD^+^	21	16	12	9	20	1	16	0	15	1
NADP^+^	6	30	5	1	5	1	20	3	22	1
PP_i_	0	36	0	0	0	0	30	3	33	0
NADPH	29	6	19	3	21	1	5	1	5	1
NADH	14	19	14	0	13	1	10	9	18	1
2-Oxoglutarate	6	19	4	2	6	0	7	11	17	1
Pyruvate	12	10	9	3	12	0	8	1	9	0
AMP	2	19	1	0	1	0	13	2	15	0
NH_3_	13	7	12	1	13	0	6	0	6	0
CoA	4	17	4	0	4	0	8	7	15	0
SAM	16	1	9	0	9	0	1	0	1	0
Acetyl-CoA	13	3	8	4	12	0	3	0	3	0
Glutamine	14	1	13	0	13	0	1	0	1	0

*Reactant and product frequency for each compound of the 402 core reactions is given and further classified into participation in exergonic/endergonic reactions for physiological and vent conditions. Number of occurrences on the left or right side of reactions can exceed numbers of reactions for which values of ΔG are obtained.*

*^a^Physiological condition is defined as pH 7 at 25°C and concentrations at 1 mM.*

*^b^Vent condition is defined as pH 9 at 80°C and nonequilibrium 1:0.01 mM reactant to product concentrations.*

The effect of H_2_ across the core is substantial, with 337–342 out of 353 (95–97%) of core reactions that deliver a value for Δ*G* being exergonic (Δ*G* ≤ 0 kJ⋅mol^−1^) under nonequilibrium conditions with H_2_ at 1 μM, 80–100°C, and pH 7–10. Under these conditions, only 12 out of 353 core reactions are endergonic by ≥5 kJ⋅mol^−1^ (Supplementary Table 8). It is noteworthy that alkalinity impacts the thermodynamics of metabolic origin because it strongly affects the electron donating potential of H_2_ (Supplementary Table 6). Modern geochemical systems synthesize formate (Lang et al., 2018) and acetate (Sherwood Lollar et al., 2021) in abiotic reactions that blueprint the CO_2_-fixing reactions of microbes.

Are the conditions that we are investigating realistic in a primordial geochemical context? We have investigated the temperature range 25 to 100°C, the pH range 1–14, and H_2_ concentrations from 1 μM to 100 mM. Those ranges span conditions existing today at the serpentinizing Lost City hydrothermal field, where the temperature range is 40–90°C, the pH is 9–11, and H_2_ concentrations range from 1 to 15 mM (Kelley et al., 2005). Are such conditions primordial? Serpentinizing systems have existed since there was liquid water on earth (Sleep et al., 2011). We observed a tendency for the largest proportion of reactions to be exergonic around pH 9, 80°C and at low H_2_ concentrations, very much in line with, but not constrained by, modern conditions at Lost City, which provide a window into conditions on the early Earth (Sleep et al., 2011; Schrenk et al., 2013). We allowed the concentration of CO_2_ to vary freely across analyses, having a substrate concentration of 1 mM under nonequilibrium conditions. In natural environments, CO_2_ and bicarbonate concentrations vary across extremes. While Lost City itself has very low inorganic carbon and CO_2_, Kelley et al. (2002) report CO_2_ concentrations in vent fluids from 3 to 215 mM, while modern sea water contains roughly 11 μM CO_2_ and 2 mM HCO_3_^–^, some modern hydrothermal systems emit pure CO_2_ gas (Steffens et al., 2021) and other submarine hydrothermal vents emit pure, supercritical CO_2_ as bubbles at 1.4 km depth and high pressure (Zhang et al., 2020). On the early earth, global CO_2_ levels were generally very high (Zahnle et al., 2007; Sossi et al., 2020), but local CO_2_ concentrations might have varied as much as they do in modern environments. In general, submarine hydrothermal systems exist under very high pressure and therefore allow gasses to dissolve up to very high concentrations, today and on the early earth.

In laboratory scale model vents (Preiner et al., 2020), a 10 bar partial pressure of H_2_ at pH 9 and 100°C corresponding to 5 mM H_2_ in solution (Supplementary Table 6) and within the range of 1–15 mM H_2_ concentrations observed at Lost City (Kelley et al., 2005), will reduce CO_2_ to formate, acetate and pyruvate, although much lower H_2_ partial pressures will also suffice for the same reaction (Preiner et al., 2020). That is, geochemical H_2_ and CO_2_ spontaneously generate central compounds of autotrophic metabolism in the acetyl-CoA pathway in the presence of metal catalysts (Preiner et al., 2020). This is noteworthy not only because of the congruence between the products of the abiotic and enzymatic products from H_2_ and CO_2_ but also because earlier studies of H_2_-dependent CO_2_ reduction under higher pressures and temperatures, but performed in inert titanium reactors in the absence of catalysts, did not detect the synthesis of either acetate or pyruvate among the products (McCollom and Seewald, 2003), whereas inclusion of iron or nickel, either as native metal or as oxide or sulfide minerals effectively replace the pathway to pyruvate, yielding physiologically relevant concentrations of pyruvate (∼40 μM) overnight. From the outset of the first abiotic reactions to the origin of an enzymatically catalyzed metabolism in LUCA, redox reactions were integral to metabolic origin, whereby H_2_ provided an ample and biochemically accessible supply of electrons throughout that process, particularly under the alkaline conditions of serpentinization (Preiner et al., 2019).

### Water

Views concerning the role of water at origins differ widely. One view has it that water is inhibitory at the origin of life because reactions that generate water, in particular polymerization reactions, proceed against the pushback of a 55 M product (Marshall, 2020). The other view is that water is essential to origins because it is both the solvent of all molecules of life and the most common reactant in microbial metabolic networks (do Nascimento Vieira et al., 2020). An underappreciated aspect of hydrothermal systems is that they harbor abundant local microenvironments of low water activity (Lamadrid et al., 2017). The serpentinization process that underpins the formation of H_2_ for CO_2_ reduction at metabolic origin entails rock-water interactions that consume about 20 molecules of H_2_O per molecule of H_2_ formed and about 100 molecules of H_2_O per molecule of abiotic methane formed from CO_2_ (Preiner et al., 2018). In the present calculations, water concentration is fixed at 55 M and cannot be changed in these calculations (Alberty, 1998; Flamholz et al., 2012). Water is furthermore the most common compound in the reactions of the core, appearing in 120 reactions, 97% of which are exergonic regardless of whether water is consumed or produced against the 55 M gradient (Table 1). From the thermodynamic perspective H_2_O exerts no inhibitory effect upon the reactions of core biosynthesis. That, and the frequency of water as a reactant (Table 1) suggest that the reactions that gave rise to LUCA’s metabolism arose in an aqueous environment, a premise preferable to the proposition that the chemistry of life began in non-aqueous environments, and only later transformed *en masse* into the aqueous reactions of the cytosol.

### Salt

Salt concentrations differ in marine vs. freshwater origin environments, and some origin of life theories posit that life arose in freshwater environments based on arguments relating to concentrations of K^+^ (Korolev, 2021) as opposed to arguments based upon reactions of carbon (Preiner et al., 2020). Seawater has an ionic strength of ca. 700 mM, while cytosol has a variable ionic strength on the order of 20–900 mM in *E. coli* (Richey et al., 1987) but exceeding 2,000 mM in some archaea (Ginzburg et al., 1970). Hydrothermal effluent has an ionic strength on the order of 20-800 mM (Kelley et al., 2002). Across the range from 0 to 2.5 M, ionic strength has very little impact on Δ*G* of core reactions as estimated by implementation of the component contribution method employed here (see Supplementary Figure 3 and Supplementary Table 5).

### Nonequilibrium Conditions Have a Pronounced but Not a Dominant Effect

Using eQuilibrator (Noor et al., 2013), water activity cannot be perturbed but is already taken into account in Δ*G*. The effect of ionic strength was small (Supplementary Figure 3 and Supplementary Table 5). To compare the effects for parameters investigated here that did show effects, we plotted the mean and range of values of Δ*G* for comparison of temperature (25°C vs. 80°C), pH (7 vs. 9), nonequilibrium vs. equal reactant and product concentrations, and organic reductants vs. H_2_. Nonequilibrium conditions have the most pronounced effect across reactions of the core (Figure 5). But even for conditions of 1 mM reactant and product concentrations, the mean of the 351 reactions that deliver an estimate of Δ*G* is still negative. For reactions that are only slightly endergonic, the effect of nonequilibrium conditions can render the value of Δ*G* negative (Figure 5).

Though nonequilibrium conditions have a pronounced effect, they do not fundamentally distort the picture for individual reactions. This is shown in Figure 4, where the estimate of Δ*G* for amino acid synthesis is compared for physiological conditions (with 1 mM reactant and product concentrations, pH 7, 25°C, gasses at 1 atm) vs. conditions more similar to those in serpentinizing hydrothermal systems (nonequilibrium with 1:0.01 mM concentrations, 1 μM of H_2_ instead of organic reductants, other gasses at 1 atm) for 111 reactions of amino acid metabolism starting from the key intermediates for the biosynthesis of the families of amino acids: pyruvate, oxalacetate, 2-oxoglutarate, phosphoenolpyruvate, 3 phosphoglycerate and C5 sugars (Supplementary Table 7). The main effect is observed for reactions that are close to equilibrium (Δ*G*≈ 0) to begin with. This indicates that there is a natural tendency for the individual reactions of amino acid metabolism from H_2_, CO_2_ and NH_3_ in the core to go forward both in physiological and vent conditions, a finding that does not follow from calculations of one-step amino acid syntheses from the same reactants (Amend and Shock, 1998; Amend et al., 2013). It is also important because amino acids are essential sources of C and N for the biosynthesis of bases and cofactors (Wimmer et al., 2021a). Note that the reactions in Figure 4 correspond to KEGG reactions and are detached from the overall metabolic network, such that the products of an upstream reaction do not necessarily generate all of the reactants required for the subsequent reaction. Despite that caveat, the general exergonic nature of the individual reactions is evident.

### Phosphate

Phosphate is a component of many of the metabolic energy currencies. It forms high energy bonds which are cleaved in exergonic reactions that, when enzymatically coupled to endergonic reactions of metabolism, allow the latter to go forward. The entry of phosphate into metabolism is a heavily debated topic. One view has it that high energy phosphorous minerals reacted with inert carbon compounds (Pasek, 2020), another view has it that inert phosphate reacted with highly reactive carbonyl groups (Martin, 2020), yet another view, based on computer simulations, is that simple protometabolic networks might have been possible without phosphate (Goldford et al., 2017, 2019), though subsequent work identified contrary effects (Tian et al., 2019). In the conserved core of microbial metabolism, LUCA’s metabolism, phosphate is indispensable. Of 402 core reactions, 260 (65%) involve phosphate or phosphorylated compounds. For comparison, 83% of the core reactions contain nitrogen. Moreover, 80 reactions (20%) involve ATP hydrolysis or phosphoanhydride hydrolysis of other nucleoside triphosphates in the biosynthetic direction. Among those NTP hydrolyzing reactions, 26 generate ADP and P_i_, 10 generate AMP and PP_i_, while 33 generate phosphorylated products (Supplementary Table 9). Furthermore, all of the cofactors that generate amino acids, bases and cofactors themselves, except biotin, contain phosphate. There can be no question that the biosynthetic core as it existed in LUCA had phosphate inextricably hard wired into its fabric.

That phosphate was part of the core and LUCA’s metabolism seems difficult to debate, but how did it enter the core? Net ATP synthesis in the core is afforded by substrate level phosphorylation involving acetyl phosphate via acetyl-CoA (Ferry and House, 2006; Martin and Russell, 2007). ATP is synthesized there by energy conserving reactions that, like thioester synthesis (Huber and Wächtershäuser, 1997; Kitadai et al., 2021), can proceed without enzymes (Sousa et al., 2018; Whicher et al., 2018). Under nonequilibrium vent conditions, the reaction of acetyl-CoA with P_i_—the reaction of phosphate with carbonyl groups—to yield acetyl phosphate is exergonic by –18.6 kJ⋅mol^−1^, the subsequent reaction of acetyl phosphate with ADP to yield ATP and acetate is exergonic by –40.1 kJ⋅mol^−1^ (Supplementary Table 8). The energetics of acetyl-CoA synthesis from H_2_, CO_2_, and coenzyme A are, however, strongly dependent upon the H_2_ partial pressure (Fuchs, 2011). Under nonequilibrium conditions, the reaction is endergonic by +37 kJ⋅mol^−1^ at 1 μM H_2_ and pH 9 but at 1 mM H_2_ it becomes exergonic by –44 kJ⋅mol^−1^. This crucial CO_2_ activating reaction requires H_2_ partial pressures corresponding to potentials on the order of –660 mV at metabolic origin, which abound in natural H_2_-producing vents (Boyd et al., 2020). At pH 9 and 100°C, –660 mV corresponds to ca. 1 atm H_2_ or 10^5^ Pa H_2_ or 560 μM H_2_ (Supplementary Table 6), less H_2_ than is found in serpentinizing systems, which contain typically 1 mM H_2_ or more, with 1–15 mmol H_2_ per kg aqueous effluent observed in the case of Lost City (Kelley et al., 2005).

Of the 351 core reactions that deliver a value of Δ*G*, 80 involve hydrolysis of anhydride bonds in ATP or other triphosphates as an energy currency (Supplementary Table 9). None of the reactions in the core utilize pyrophosphate (PP_i_) as an energy source, but 36 reactions generate PP_i_ from nucleoside triphosphates (Table 1). In contrast to many traditional views, PP_i_ was not a source of energy in early metabolism (Wimmer et al., 2021b). If we subtract the contribution of phosphoanhydride hydrolysis from those 80 reactions, 63 become endergonic by more than 20 kJ⋅mol^−1^ (Supplementary Table 9), a very steep energetic barrier, even under nonequilibrium vent conditions. High energy phosphate bonds are thus essential integral components of the core, apparently as old as metabolism itself and likely the result of inert phosphate reacting with carbonyl groups generated as intermediates of CO_2_ reduction. The pressing question remains, however: What is the driving force behind ∼75% of the core reactions that are exergonic independent of ATP?

### The Dark Energy at Origins Resides in Carbon

Because our starting compounds are H_2_, CO_2_, NH_3_, H_2_S, H_2_O, and P_i_ (Figure 1 and Supplementary Table 1), because no other sources of energy are introduced into the system, and because no N–N or O–O bonds are formed in the core, reactions of carbon are the only candidate for the source of free energy change in core reactions without ATP. We identified 10 organic reaction types that together account for half of ATP-independent exergonic reactions (Table 2). Among the 351 reactions that deliver values of Δ*G*, 10 involve S-adenosylmethionine dependent alkyl transfers (Δ*G’* = −24 kJ⋅mol^−1^; Lewis and Wolfenden, 2018). Six reactions involve folate dependent alkyl transfers (Δ*G’* = −30 kJ⋅mol^−1^; Thauer et al., 1977) or acyl transfers (Δ*G’* = −26 kJ⋅mol^−1^; Decker et al., 1970). Acyl thiol ester (thioester) hydrolysis (Δ*G’* = −32 kJ⋅mol^−1^; Buckel and Eggerer, 1965) drives 14 reactions and acyl phosphate hydrolysis (Δ*G’* = −45 kJ⋅mol^−1^; Decker et al., 1970) drives four reactions (Supplementary Table 10).

**TABLE 2:** Energy release in the core.

Gibbs energy changes for exergonic carbon based reactions in the core			
Reaction	*N*	Δ*G*^a^	References
Pyruvate formation from H_2_+CO_2_	1	−57	Preiner et al. (2020)
Ring formations	35	−10 to −25	Goldberg and Tewari (1989)
Decarboxylations	30	−20	Dimroth and Schink (1998)
SAM dependent alkyl transfers	10	−24	Lewis and Wolfenden (2018)
Folate dependent acyl transfers	2	−26	Decker et al. (1970) ^b^
Reductions	44	−28	
Folate dependent alkyl transfers	4	−30	Thauer et al. (2008)
Acyl thiol ester hydrolyses	14	−32	Buckel and Eggerer (1965)
Acyl phosphate hydrolyses	4	−45	Decker et al. (1970)
Aromatic formation	31	−60 to −150	Morrison and Boyd (1977)

*Estimated ΔG for different reaction types. N is the number of reactions among 351 reactions in the core for which values of ΔG are obtained.*

*^a^ΔG [kJ⋅mol^−1^] as given in references.*

*^b^Average ΔG for 44 NAD(P) H-, ferredoxin-, and formate-dependent reductions in the core under conditions specified in Supplementary Table 10.*

The only input compound that is reduced in reactions of the core is CO_2_ (Figure 1). For the 44 reactions involving reductions of carbon with reduced nicotinamide, flavin, ferredoxin or formate, reactions that are exergonic under physiological conditions (Decker et al., 1970; Thauer et al., 1977), the average Δ*G* in the core is –28 kJ⋅mol^−1^ under nonequilibrium 1:0.01 mM conditions at 80°C and pH 9 (Table 2). Decarboxylations, with a Δ*G*°’ on the order of –20 kJ⋅mol^−1^ (Dimroth and Schink, 1998) occur in 30 reactions, 10 of which are oxidative decarboxylations (Supplementary Table 10). In addition, many reactions of the core generate aromatics from non-aromatic substrates. Aromaticity entails very large changes in Δ*G*, on the order of –60 to –150 kJ⋅mol^−1^ or more (Morrison and Boyd, 1977). The amino acids, bases and cofactors produced by the core involve the synthesis of 31 aromatic rings and 35 ring closure reactions (Goldberg and Tewari, 1989) that are involved in their formation.

Including the exergonic synthesis of pyruvate from H_2_ and carbon dioxide (Preiner et al., 2020), these sources of carbon-based energy (Table 2) contribute to favorable thermodynamics in 50% of core reactions (175/351), more than twice the number of reactions (80/351) driven by ATP hydrolysis, though sometimes with a smaller contribution to Δ*G* per reaction. The core’s remaining 84 exergonic conversions (24%) are driven by other energy releasing reactions of carbon that do not fall into the 10 categories listed in Table 2. At the energetic extremes, only 12 reactions in the core (3%) are endergonic by >5 kJ⋅mol^−1^ under nonequilibrium conditions at 80°C and pH 9 (Supplementary Table 8). The most highly exergonic reaction in the core is catalyzed by pyridoxal phosphate synthase, the mechanism of which (Laber et al., 1999) requires no ATP and eliminates 3 H_2_O against a 55 M gradient but with a Δ*G* of −383 kJ⋅mol^−1^ (Supplementary Table 8) because of the reaction product’s aromaticity relative to its reactants. In the simplest interpretation, the carbon-based sources of energy shown in Table 2 are identical to the sources of energy that gave rise to metabolism, which in turn gave rise to LUCA. The overall flow of energy through the core from high energy substrate H_2_ plus low energy CO_2_ to reactive carbon compounds and its thermodynamically more stable products is schematically summarized in Supplementary Figure 5.

## Conclusion

The individual biochemical reactions underpinning the synthesis of amino acids, nucleotides and cofactors in modern cells trace to LUCA because of their universality. These reactions are exergonic under the conditions of H_2_-producing geochemical systems, where formate (Lang et al., 2010), acetate (Sherwood Lollar et al., 2021) and methane (Proskurowski et al., 2008) are synthesized in abiotic reactions today. In the present work, we have not investigated the role of high hydrostatic pressure exerted by the water column in deep water. This is because the tool we employed to estimate values of Δ*G* through the component contribution method is designed for studies of microbial metabolism at ambient pressures. At higher hydrostatic pressures, as are found in hydrothermal vents (Kelley et al., 2002), a shift in equilibria toward the formation of more products for reactions of the type A + B → C might be expected according to Le Chatelier’s principle. However, it is noteworthy that autotrophic microbes isolated from hydrothermal vents at depths of 2.4 km (ca. 240 bar hydrostatic pressure) grow well under ambient pressure (Beatty et al., 2005), such that in the presence of excellent catalysts (enzymes), high pressure might not be a decisive factor whereby in the presence of only mineral catalysts, hydrostatic pressure might play an important role. Indeed, gasses are compressed considerably at 240 bar and dissolve better in water so that reactant concentrations of dissolved gasses are higher than at ambient pressure. On the contrary, liquid water is compressed only very little (<1% at 240 bar) so that microbes without gas inclusions stay essentially untouched. Methanogens that lack cytochromes require only 10^–4^ to 10^–5^ atm of H_2_ for growth (Thauer et al., 2008). Like acetogens, their main energy harnessing reaction results in the conversion of about 20 molecules of CO_2_ into waste product (methane for methanogens and acetate for acetogens) for every molecule of CO_2_ that is incorporated into cell mass (Martin, 2020). That is, cell mass, the product of metabolism, is just a byproduct of the main energy releasing reaction of the cell. The environment where metabolism arose must therefore have harbored a constantly out of equilibrium supply of carbon, electrons, and transition metal catalysts to promote energy releasing reactions. Reactions of H_2_ and CO_2_ in serpentinizing hydrothermal systems fulfill those criteria (Schrenk et al., 2013) in a manner that directly connects to the metabolism of modern cells (Preiner et al., 2018; Xavier et al., 2020).

The present data uncover a hitherto unique thermodynamic link between core biochemistry as a whole and the conditions of a geochemical environment known to have existed on the early Earth. The reactions of the core require neither membrane proteins, cytochromes, quinones, nor light. Their thermodynamics indicate that the core biosynthetic reactions of microbial metabolism could have arisen from soluble (Martin and Russell, 2007; Muchowska et al., 2019) and surface-catalyzed (Wächtershäuser, 1988; Preiner et al., 2020) reactions in the dark, under hot, aqueous, H_2_-bearing geochemical environments, independent of exposed land masses (light) or the existence of water with a low ionic strength. Though ATP provides energy for roughly one fourth of the core’s reactions, a three fourth’s majority of reactions derive their energy release from reactions of carbon compounds germane to metabolism itself, sources of chemical energy that, with the exception of thioesters (Semenov et al., 2016) and acyl phosphates (Martin and Russell, 2007; Whicher et al., 2018), have escaped the focus of previous investigations into early metabolic evolution. While estimates of Δ*G* are, of course, silent on reaction rates, activation energy, and catalysts (Wolfenden, 2011), the crucial energetic role of hydrogen (Thauer et al., 2008; Fuchs, 2011; Amend et al., 2013; Boyd et al., 2020; Preiner et al., 2020) and the exergonic biochemical reactions of carbon reported here uncover a natural thermodynamic tendency for the individual reactions of metabolism to arise from H_2_, CO_2_, NH_3_, and H_2_S in the presence of phosphate.

## Data Availability Statement

The original contributions presented in the study are included in the article/Supplementary Material, further inquiries can be directed to the corresponding author/s.

## Author Contributions

WM and JW: conceptualization and visualization. JW, WM, FS, KK, and JX: methodology. JW, JX, and WM: data curation. JW, WM, AV, DP, JL, MP, and KK: formal analysis. WM: writing—original draft, supervision, and funding acquisition. WM, JW, JX, AV, DP, JL, FS, KK, and MP: writing—review and editing. All authors contributed to the article and approved the submitted version.

## Conflict of Interest

The authors declare that the research was conducted in the absence of any commercial or financial relationships that could be construed as a potential conflict of interest.

## Publisher’s Note

All claims expressed in this article are solely those of the authors and do not necessarily represent those of their affiliated organizations, or those of the publisher, the editors and the reviewers. Any product that may be evaluated in this article, or claim that may be made by its manufacturer, is not guaranteed or endorsed by the publisher.
