# Supplementary material for: Energy at Origins: Favorable Thermodynamics of Biosynthetic Reactions in the Last Universal Common Ancestor (LUCA)
Source: Front Microbiol. 2021 Dec 13;12:793664. doi: 10.3389/fmicb.2021.793664 (PMC8710812; doi:10.3389/fmicb.2021.793664)
Supplement: Supplementary file 11 [file Data_Sheet_1.pdf]

Supplementary Material

Contains ten tables and five figures.

Supplementary Table 1. Biosynthetic core comprising 402 metabolic reactions which are polarized in the direction of cell synthesis and needed to synthesize cofactors, bases and amino acids from H_2, CO_2, NH_3, P_i, H_2O and H_2S. The reactions are given in KEGG format. The synthesis in which they participate is indicated as well as the associated KEGG Module, Enzyme Commission number and KEGG Orthology identifier.

Supplementary Table 2. Comparison of Gibbs energy with literature. Gibbs energy for reactions in the biosynthetic core involved in CO_2 fixation are compared to Fuchs, 2011.

Supplementary Table 3. Gibbs energy for 402 biosynthetic core reactions across pH 1−14 and temperature 25−100 °C. The ionic strength is constant at 250 mM. The pH was altered from 1−14 and temperature from 25−100 °C. The concentrations are (**A**) 1 mM for all compounds, (**B**) reactant:product ratio 1:0.1 mM, (**C**) 1:0.01 mM, (**D**) 1:0.001 mM, (**E**) 1:0.0001 mM and (**F**) 1:10 mM. Gibbs energies are coloured gradually, from exergonic in red to endergonic in blue.

Supplementary Table 4. Gibbs energy for 73 biosynthetic core reactions at different hydrogen concentrations after replacing natural reductants with H_2. The temperature was altered from 25−100 °C and pH from 1−14 at constant ionic strength 250 mM. Concentration ratios are at nonequilibrium 1:0.01 mM. Hydrogen concentration was fixed to (**A**) 0.001 mM, (**B**) 0.01 mM, (**C**) 0.1 mM, (**D**) 1 mM, (**E**) 10 mM and (**F**) 100 mM.

Supplementary Table 5. Gibbs energy for 402 biosynthetic core reactions at different ionic strengths. The temperature was altered from 25−100 °C and pH from 1−14. Reactions where reductants were replaced with H_2 are included. Hydrogen concentration is fixed to 1 µM with the remaining compounds at nonequilibrium 1:0.01 mM. Ionic strength was varied to (**A**) 2.5 mM, (**B**) 25 mM, (**C**) 250 mM and (**D**) 2.5 M.

Supplementary Table 6. Electrochemical potential: H_2 pressure, temperature and pH dependency. Conversion of H_2 partial pressures and H_2 concentrations with associated midpoint potentials in solution at different temperatures.

Supplementary Table 7. Synthesis pathways for 20 canonical amino acids. The syntheses start from key intermediates pyruvate, oxalacetate, 2-oxoglutarate, phosphoenolpyruvate, 3-phosphoglycerate and C5 sugars according to Martin, 2020. Alternative reactions and pathway branches are indicated. Additional synthesis pathways from KEGG are not included but retained in the biosynthetic core dataset.

Supplementary Table 8. Biosynthetic core reactions ranked by associated Gibbs energy in extreme hydrothermal vent conditions. The 402 reactions are sorted in decreasing order by ΔG. Reactions where reductants were replaced with H_2 are included. Hydrogen concentration is fixed to 1 µM with the remaining compounds at nonequilibrium 1:0.01 mM. Ionic strength is at 250 mM.

Supplementary Table 9. Reactions involving ATP hydrolysis as an energy source with calculable Gibbs energy in vent conditions. The original value of ΔG is shown as well as after subtraction of the corresponding ΔG for ATP hydrolysis. For phosphorylation reactions, the energetic value of ATP hydrolysis to ADP + P_i was subtracted. For diphosphorylation reactions, the energetic value of ATP hydrolysis to AMP + PP_i was subtracted. For the remaining reactions, the average of both energetic values was subtracted, indicated by asterisks.

Supplementary Table 10. Energy types among 351 biosynthetic core reactions with calculable Gibbs energy in hydrothermal vent conditions. Gibbs energy was determined for 80 °C, pH 9 temperature and constant ionic strength 250 mM. Concentration ratios are at nonequilibrium 1:0.01 mM. No reductants were replaced. The reactions are ranked by their Gibbs energy. Energy sources correspond to ATP* (ATP or NTP hydrolysis), folate acyl/alkyl (folate dependent acyl/alkyl transfers), SAM (S-adenosyl-methionine dependent alkyl transfers), reduction (H_2, NAD(P)H, ferredoxin, flavodoxin or formate dependent), decarboxylation (generating CO_2), acyl phosphate (acyl phosphate hydrolysis), thioester (CoA ester hydrolysis). Phosphoenolpyruvate is not counted as an energy source as it is derived from ATP in the core. The contribution of nonequilibrium is not counted as an energy source.

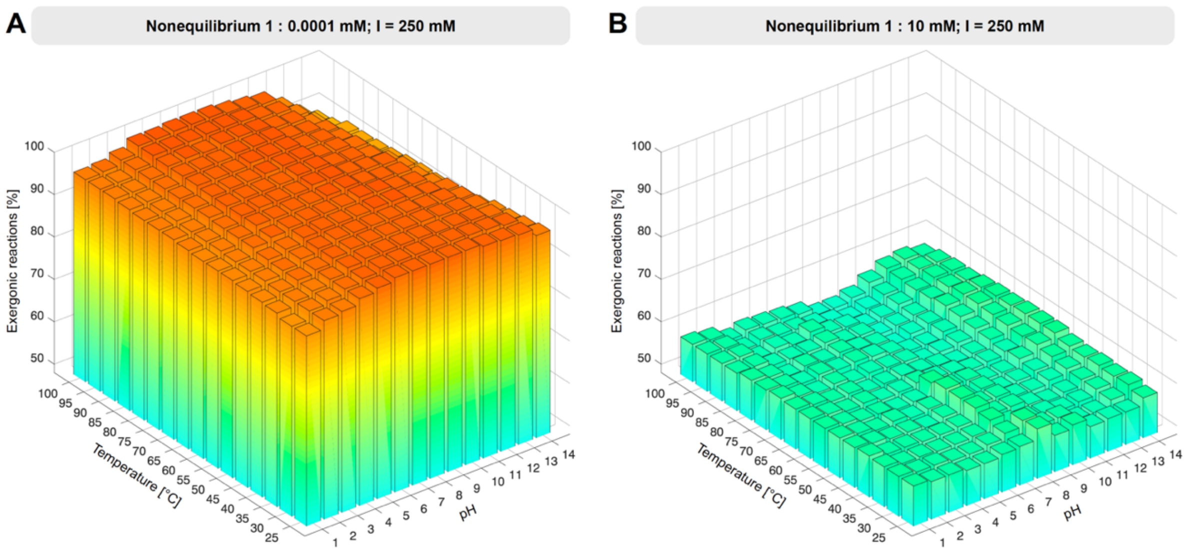

Supplementary Figure 1. Effect of nonequilibrium conditions (1:0.0001 and 1:10 mM). Proportion of exergonic reactions among 351 reactions for which Δ*G* was obtained. Ionic strength is constant at 250 mM for a concentration ratio at **(A)** nonequilibrium 1:0.0001 mM (reactant excess) and **(B)** nonequilibrium 1:10 mM (product excess).

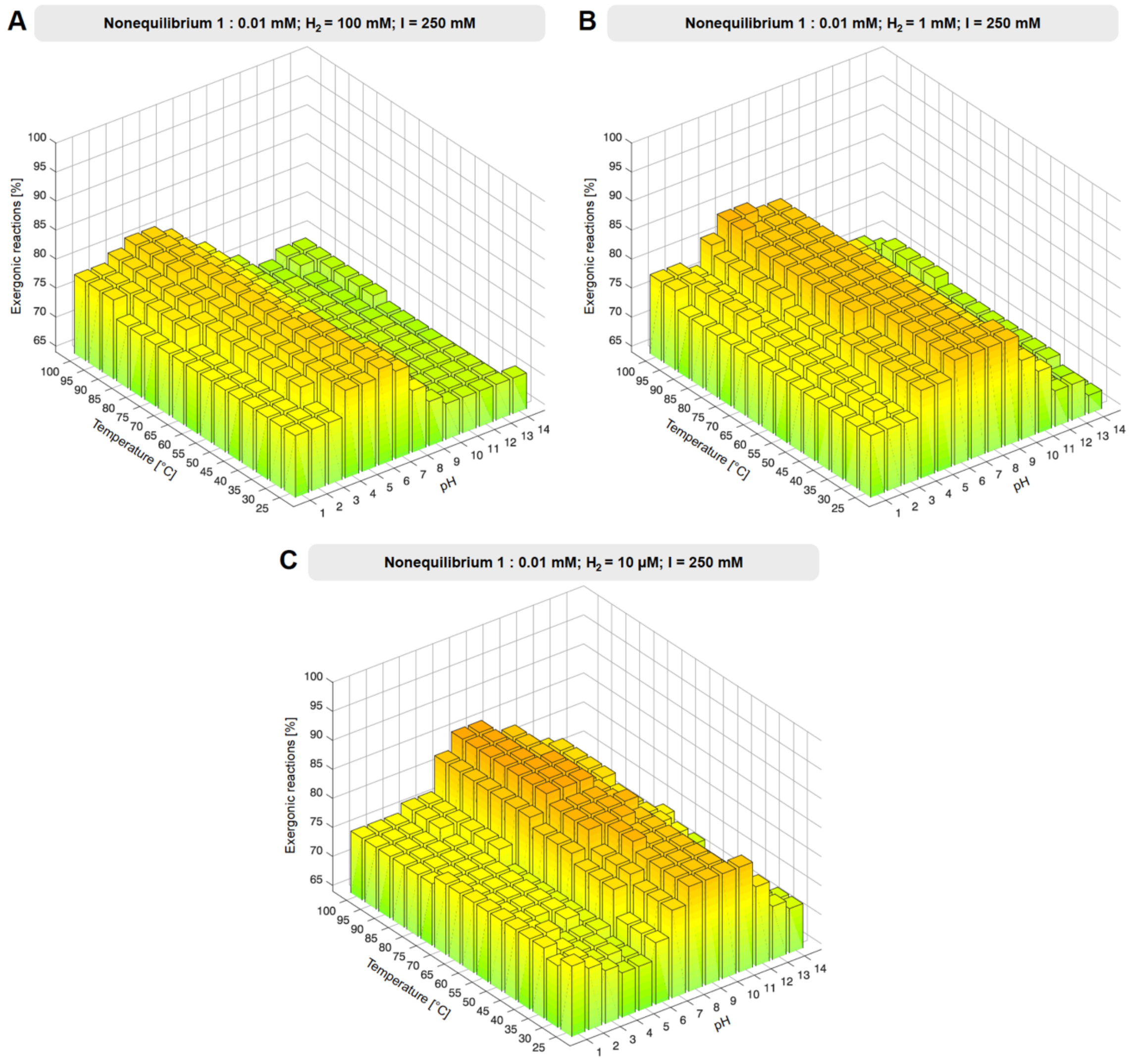

Supplementary Figure 2. Effect of environmental H_2. Proportion of exergonic reactions at **(A)** 100 mM, **(B)** 1 mM and **(C)** 10 µM H_2 as reactant and product. Concentration ratios are 1:0.01 mM for reactants other than H_2, with constant ionic strength, I. Bars indicate the proportion of exergonic reactions among the 67 redox reactions of the core that yield values of ΔG with these parameters.

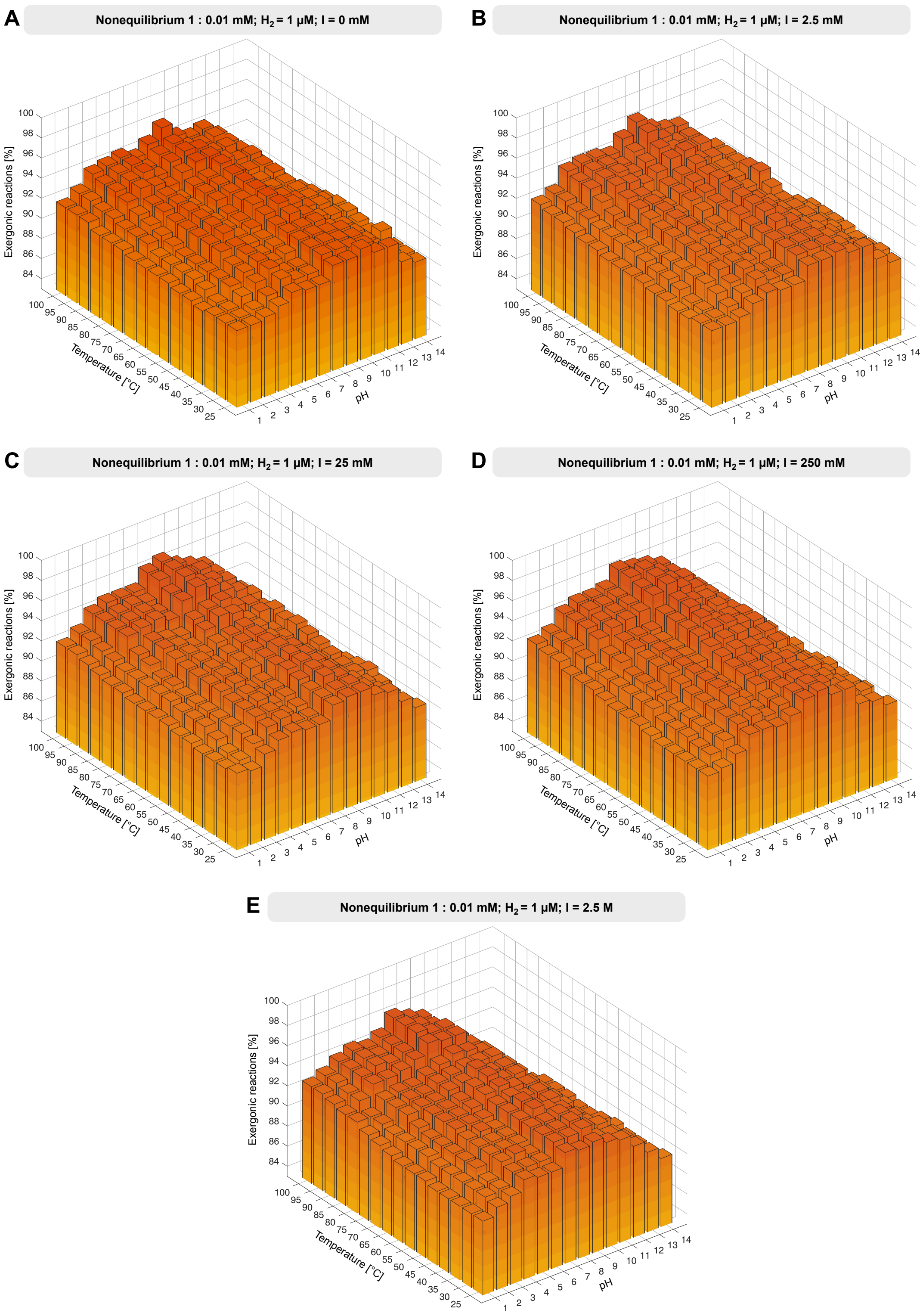

Supplementary Figure 3. Effect of ionic strength. Proportion of exergonic reactions at **(A)** 0 mM, **(B)** 2.5 mM, **(C)** 25 mM, **(D)** 250 mM and **(E)** 2.5 M ionic strength. Concentration ratios are at nonequilibrium, 1:0.01 mM, with constant H_2 concentration 1 µM. Bars indicate the proportion of exergonic reactions among the 353 reactions of the core, including reductant substituted reactions, that yield values of ΔG.

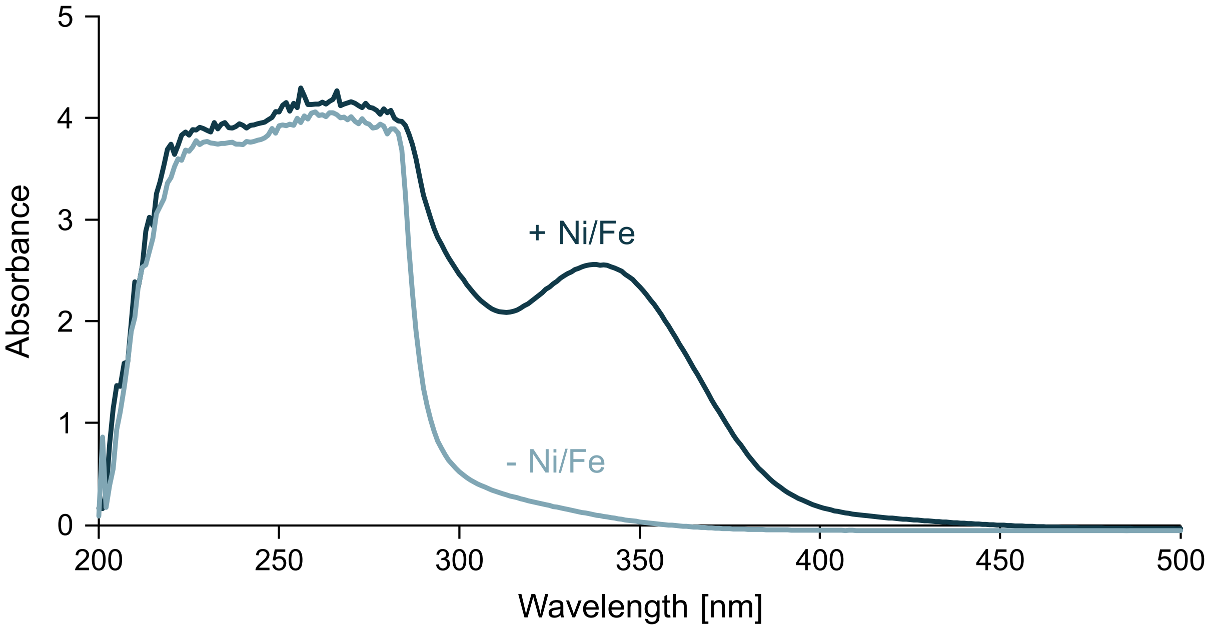

Supplementary Figure 4. NAD^+ reduction with H_2 catalyzed by metals. Reduction of NAD^+ to NADH with H_2 under alkaline conditions (pH 8.5), see Methods. The control experiment (light curve) performed without metal catalyst shows no increase in NADH concentration. The reaction including iron and nickel powder as catalysts (dark curve) shows NADH accumulation observed as an increase of absorbance at 339 nm.

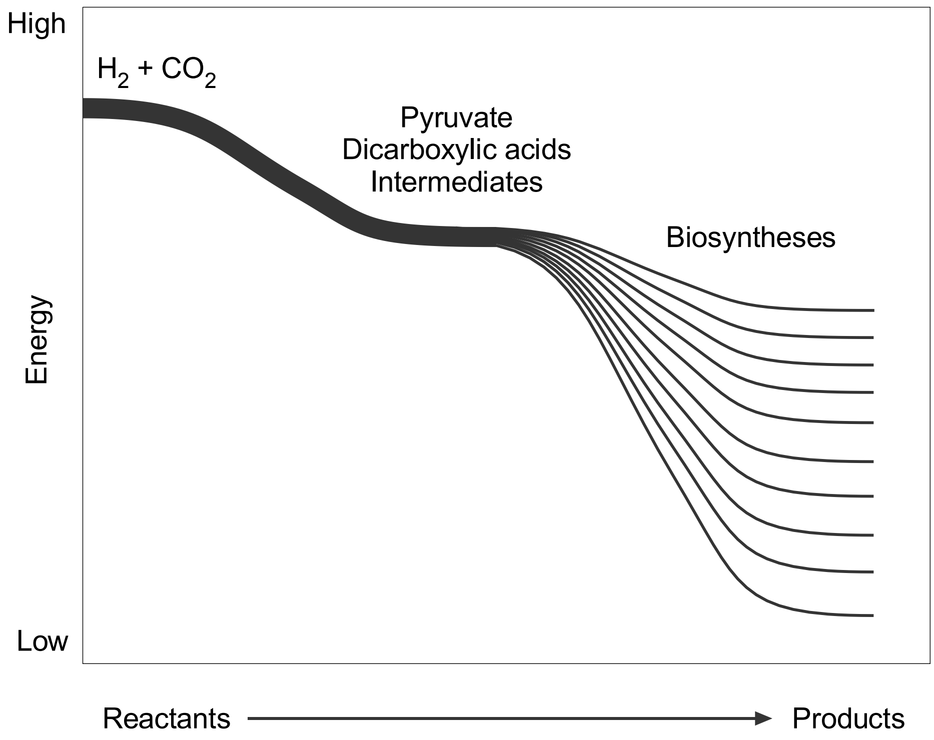

Supplementary Figure 5. A schematic diagram showing the flow of energy from more reactive compounds to more stable compounds in the reactions of the core starting from H_2 and CO_2 and progressing towards amino acids, bases and cofactors. For simplicity nitrogen is not indicated. The synthesis of reduced organic compounds via pyruvate and other key intermediates releases energy and generates more stable products. The y axis is generically designated energy, but would correspond to heat of combustion for many compounds (Morowitz et al., 2000). The figure is adapted from Fig. 1.1 of Richter (1998).

Supplementary References

Morowitz, H. J., Kostelnik, J. D., Yang, J., and Cody, G. D. (2000). The origin of intermediary metabolism. *Proc. Natl. Acad. Sci. U. S. A*. 97, 7704–7708. doi: 10.1073/pnas.110153997

Richter, G. (1998). *Stoffwechselphysiologie der Pflanzen*. Thieme Verlag, Stuttgart.
